# Muscle Lim Protein (MLP)/CSRP3 at the crossroad between mechanotransduction and autophagy

**DOI:** 10.1038/cddis.2015.308

**Published:** 2015-10-22

**Authors:** M M Rashid, A Runci, M A Russo, M Tafani

**Affiliations:** 1 Department of Experimental Medicine, University of Rome, Sapienza, Rome, Italy; 2 Department of Cellular and Molecular Pathology, IRCCS San Raffaele, Rome, Italy

The ongoing molecular investigation of the basic contractile units of the sarcomere in striated muscles have revealed a highly complex system composed of a large number of proteins each one with a specific task.^1^ However, a growing number of studies is pointing the attention to the fact that many of these structural proteins may also be involved in pathways and functions other than contraction but always related to muscle cell maintenance, repair, differentiation, signaling, etc.^1^ In this context an important role has been attributed to the muscle LIM protein (MLP). MLP is a member of the cysteine and glycine rich protein (CSRP) family that is composed by CSRP1, CSRP2 and CSRP3 or MLP. CSPRs, in turn, belong to the larger family of LIM domain proteins.^2^ MLP is expressed in striated muscle at the level of sarcomere, the intercalated disk and the costamere where it interacts with telethonin, *α*-actinin, cofilin-2 and calcineurin among others and contains two LIM domains that are zinc finger domains composed of ~55 aminoacids with 8 highly conserved residues, mostly cysteine and histidine that are located at defined intervals.^2^ Through the LIM domain, MLP can modulate protein interaction and formation of macromolecular structures. Another peculiarity of MLP is that it can shuttle from the cytosol to the nucleus where it serves as a scaffold protein to adapt transcription factors to their DNA-binding sequences.^2, 3^ In fact, MLP is involved myocyte differentiation by activating transcription factors such as MyoD, myogenin, etc.^4^ To this effect, mutations in the N-terminal region of MLP have been linked to the development of dilated cardiomyopathy (DCM) or hypertrophic cardiomyopathy (HCM). Moreover, MLP protein levels or intracellular localization are altered in skeletal myopathies, such as fascioscapulohumeral muscular dystrophy, nemaline myopathy and limb girdle muscular dystrophy type 2B.^5, 6^ Therefore, the current dogma foresee an important role for MLPin the maintenance of muscle cytoarchitecture as well as in the differentiation of muscle cells. However, the cellular and molecular mechanism(s) through which MLP carries out its function is (are) still obscure.

Using a myoblast cell line we have recently demonstrated that MLP uses macroautophagy to control differentiation of myobalsts into myotubes as well as survival of myotubes in the presence of a cell death stimulus.^7^ In particular, we have used MLP silenced myoblasts to show an impairment of autophagy that, in turn, results in decreasedmyotubes formation and decreased resistance to cell death. However, the question was: how can MLP control autophagy? We provided evidences that MLP interacts and associates with LC3, a widely used autophagy marker with a crucial role during autophagosome formation. Interestingly both MLP and LC3 are microtubule associated proteins. Therefore, besides controlling muscle cell differentiation by modulating the expression of MyoD1 and myogenin mRNA, we also show that cytosolic MLP is important for the correct assembly and flux of the autophagic machinery. In fact, MLP silencing not only decreased LC3-II accumulation but also resulted in impaired degradation of long-lived proteins. Interestingly, morphological analysis of myotubes lacking MLP expression by transmission electron microscopy revealed the accumulation of damaged mitochondria and multi-membranous structures.Of note, the same multi-membranous structures with sarcomere disorganization have been described in skeletal muscles from Atg7^–/–^ and Atg5^−/−^ mice through the accumulation of damaged organelles and material. The final phenotype of these mice is characterized by muscle atrophy and features of myopathy.^8^ In fact, increasing evidences suggest that autophagy has an important physiological role during development, differentiation and muscle mass maintenance.^9^

Autophagy has also been linked to another physiological pathway such as apoptosis. Like autophagy, apoptosis has a fundamental role during development and differentiation. In fact, autophagy and apoptosis uses common proteins and it is widely accepted that increased autophagy represent a protective mechanism from apoptotic cell death. Such knowledge derives from oncology in which cancer cells increases resistance to chemotherapy-induced apoptosis by increasing autophagic flux and removal of damaged organelles and proteins.^10^ Our results show that reduced MLP expression with reduced autophagy, increases sensitivity of myotubes to apoptogens and cell death. This suggests that autophagy may represent a mechanism through which muscle cells can recover from a stress or a sub-lethal damage that do not kill the cell but requires extensive remodeling of internal structures.

Our results shed new light on the MLP and on its central role for muscle cell differentiation and remodeling. As shown in Figure 1, in skeletal muscle cells, MLP can be activated by exogenous signals such as mechanical, toxic, growth factors, etc. Once activated, MLP can move to the nucleus and/or remain in the cytosol where associates with LC3 to control the autophagic process. In this way, as nuclear MLP binds to myogenic transcription factor, thereby activating *de novo* synthesis of structural genes, cytosolic MLP regulates autophagy thereby controlling recycling of organelles and proteins and providing building blocks for muscle cell cytoarchitectural remodeling.

In conclusion, even if mutations in MLP have been linked to DCM and HCM and deregulation of MLP to skeletal muscle diseases, new questions arise. For example: which are the pathogenic mechanisms activated by the lack or inactivation of MLP? Can MLP be used as a therapeutic target? The identification of the mechanism(s) of action of MLP may drive the development of new strategies for increasing skeletal muscle cell survival and remodeling.

## Figures and Tables

**Figure 1:**
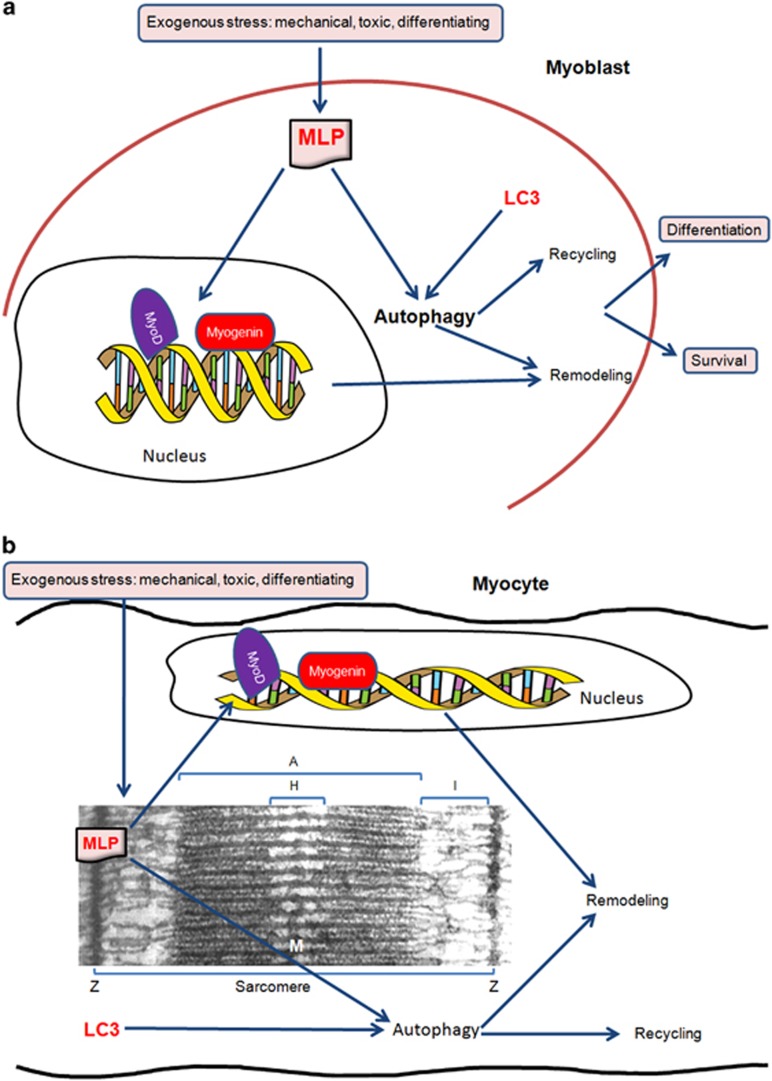
Multitasking properties of MLP. Exogenous stimuli activate MLP that, from one side, translocates to the nucleus where it binds to myogenic transcription factors and, from the other side, controls autophagy and recycling of damaged or disfunctional organelles and proteins. Both nuclear and cytosolic functions of MLP converge to differentiation and survival in myoblasts (**a**) and to remodeling in myocytes (**b**)
